# Nanoscale characterization of drug-induced microtubule filament dysfunction using super-resolution microscopy

**DOI:** 10.1186/s12915-021-01164-4

**Published:** 2021-12-11

**Authors:** Ashley M. Rozario, Sam Duwé, Cade Elliott, Riley B. Hargreaves, Gregory W. Moseley, Peter Dedecker, Donna R. Whelan, Toby D. M. Bell

**Affiliations:** 1 School of Chemistry, Monash University, Clayton, 3800 Australia; 2 Biomedical Research Institute, Hasselt University, 3590 Diepenbeek, Belgium; 3 Department of Microbiology, Monash Biomedicine Discovery Institute, Clayton, 3800 Australia; 4 Department of Chemistry, KU Leuven, 3001 Leuven, Belgium; 5 La Trobe Institute for Molecular Science, La Trobe University, Bendigo, 3552 Australia

**Keywords:** Super-resolution, Microtubules, Filament curvature, Live cell imaging, *d*STORM, SOFI, Antimitotic, Colcemid

## Abstract

**Background:**

The integrity of microtubule filament networks is essential for the roles in diverse cellular functions, and disruption of its structure or dynamics has been explored as a therapeutic approach to tackle diseases such as cancer. Microtubule-interacting drugs, sometimes referred to as antimitotics, are used in cancer therapy to target and disrupt microtubules. However, due to associated side effects on healthy cells, there is a need to develop safer drug regimens that still retain clinical efficacy. Currently, many questions remain open regarding the extent of effects on cellular physiology of microtubule-interacting drugs at clinically relevant and low doses. Here, we use super-resolution microscopies (single-molecule localization and optical fluctuation based) to reveal the initial microtubule dysfunctions caused by nanomolar concentrations of colcemid.

**Results:**

We identify previously undetected microtubule (MT) damage caused by clinically relevant doses of colcemid. Short exposure to 30–80 nM colcemid results in aberrant microtubule curvature, with a trend of increased curvature associated to increased doses, and curvatures greater than 2 rad/μm, a value associated with MT breakage. Microtubule fragmentation was detected upon treatment with ≥ 100 nM colcemid. Remarkably, lower doses (< 20 nM after 5 h) led to subtle but significant microtubule architecture remodelling characterized by increased curvature and suppression of microtubule dynamics.

**Conclusions:**

Our results support the emerging hypothesis that microtubule-interacting drugs induce non-mitotic effects in cells, and establish a multi-modal imaging assay for detecting and measuring nanoscale microtubule dysfunction. The sub-diffraction visualization of these less severe precursor perturbations compared to the established antimitotic effects of microtubule-interacting drugs offers potential for improved understanding and design of anticancer agents.

**Supplementary Information:**

The online version contains supplementary material available at 10.1186/s12915-021-01164-4.

## Background

Microtubules (MTs) form part of the cytoskeleton and have many essential roles in the cell, including maintaining cell shape and supporting the transport of organelles and vesicles [1]. To fulfil these roles, MTs form intracellular networks comprising 25-nm-wide hollow filaments, formed as polymers of *αβ*-tubulin dimers. Additionally, MT dynamics effected through tubulin assembly and disassembly contribute forces to segregate chromosomes during mitosis [2, 3]. These critical functions make MTs vulnerable to bacterial pathogens [4] and have been shown to be susceptible to viral subversion [5, 6]. The importance of MT function also presents as a viable target for cancer therapy [7, 8], and some compounds approved as anticancer drugs (paclitaxel [9] and vinblastine [10]) are known to bind tubulin and alter MT filament stability. Colchicine, a naturally occurring compound [11], induces MT depolymerization and is used to treat inflammatory diseases including gout and familial Mediterranean fever [12]. The use of colchicine, however, is limited by its toxicity and poorly defined dosing thresholds between non-toxic, toxic and lethal [13–15]. It can cause neurotoxicity [16] and is associated with renal and liver failure in cases of colchicine poisoning [13]. Despite these outcomes, colchicine still presents some potential as an anticancer compound where low concentration doses reduce the proliferation of cholangiocarcinoma cell lines, as well as decrease tumour size in mouse models [17]. Several synthetic derivatives of colchicine are being clinically tested and show promise to treat cancers in the future [18].

The physiological outcomes of treatment with MT-interacting drugs vary between patients, making it difficult to standardize a therapeutic dose for cancer treatment. This complication can be due to the individual’s resistance toward the drug, manifested either by cancer-driven genetic changes or acquired resistance over several drug treatments [19]. The onset of adverse outcomes may also be due to the drug’s lack of specificity for cancer cells, meaning non-cancerous cells also suffer some effects. MT-interacting drugs are well-established as being able to arrest cells in mitosis, preventing normal division and potentiating apoptosis. Antimitotic effects also include mitotic structural aberrations such as the formation of multiple spindles and defective daughter cells with atypical amounts of genetic information [20]. However, an emerging hypothesis to account for the action of MT-interacting drugs proposes that non-mitotic effects are key to their efficacy [21–26]. This is increasingly important for understanding therapeutic mechanisms because these drugs bind tubulin regardless of cellular phase. As a result, MT structure and dynamics become altered, affecting MT-dependent functions including intracellular transport, signalling cascades and cell motility. This may also trigger the dysfunction of MT-associated cellular components such as mitochondria [27] and actin [28]. Given the importance of MTs in the physiology of normal cells, any form of MT dysfunction will undoubtedly have significant consequences for cell health and, by extension, patient health. Ideally, any MT-interacting chemotherapeutic should be administered at the lowest dose that provides effective treatment in order to minimize off-target damage. Historically, these drugs have been studied predominantly for their antimitotic outcomes (mitotic arrest, daughter cell mutations). However, these effects are typically lethal for cells and better represent severe toxicities from high drug doses. It is increasingly clear that the much lower doses relevant in clinical use may not induce significant mitotic effects and that their mechanism of action instead affects non-mitotic MT function. These are inherently more subtle than antimitotic effects and have proven difficult to characterize using conventional imaging and biochemistry. Therefore, we set out to better understand clinically relevant mechanisms of MT-interacting drugs using complementary super-resolution microscopies which enable both sub-diffraction and dynamic live-cell observations of MT structure and function.

Super-resolution techniques build on fluorescence microscopy which has long been a cornerstone tool for visualizing intracellular features, albeit with a limited imaging resolution of ~ 200–300 nm due to the diffraction of light. In order to observe the sub-diffraction architecture of the MT network, super-resolution methods are necessary [29]. Single-molecule localization (SML) approaches such as *d*STORM (direct stochastic optical reconstruction microscopy) achieve as good as 20-nm resolution, imparting a 10-fold improvement over the conventional limit of optical microscopy [30]. While SML achieves excellent resolution gain, it is not well-suited to imaging live-cell dynamics because of the typical need for long acquisition times and high laser powers. The development of super-resolution imaging techniques that are less demanding in these regards, such as lattice light-sheet microscopy [31] and structured illumination microscopies [32] have enabled studies of live cell dynamics, however require specialized and relatively complex optical hardware. Super-resolution optical fluctuation imaging (SOFI) [33, 34] has also proven to be compatible for live-cell imaging because of comparatively mild preparation and acquisition conditions, achieving sub-diffraction resolutions through a post-processing approach, using statistical analysis of the fluorescence dynamics of the fluorophores. Like *d*STORM, SOFI can be conveniently performed on conventional wide-field microscopes with EM-CCD or sCMOS detectors. SOFI employs reversibly switching fluorescent proteins (RSFPs) such as Dronpa [35] that photoswitch in response to low laser excitation (tens of mW/cm^2^) and entails much shorter acquisition periods (seconds to tens of seconds) for individual frame generation. Though the resolution gain is, in principle, unlimited, the realities of RSFP labelling density, switching kinetics and photostability have so far restricted the practical efficiency of SOFI to an overall 4-fold resolution improvement at best [36]. Used in parallel, *d*STORM and SOFI complement each other providing super-resolution detail and live-cell relevance and context, respectively, for a holistic perspective of the biological system in question [37].

Here, we applied *d*STORM and SOFI to visualize initial MT perturbations caused by short exposure to low concentrations of colcemid. Colcemid is a derivative of colchicine commonly used in laboratory settings to arrest cells in metaphase for karyotyping assays [38, 39]. Colcemid and colchicine are both known to be MT destabilizers and share a similar IC_50_ of ~ 2.4–2.6 μM to inhibit tubulin polymerization [40, 41] when measured outside the cell. While colchicine remains a classic MT-destabilizing compound with some approved therapeutic applications, its effect on neurotoxicity limits its use for treating cancer. Modern research for colchicine-based cancer therapies focuses on developing synthetic variants, such as colcemid, that are less toxic and have greater potential for treating cancer in the future [18]. As such, our work investigating colcemid instead of colchicine is in line with this approach. With super-resolution imaging of MTs in HeLa cells, we detected significantly altered filament curvature following 5-h treatments with colcemid concentrations as low as 7 nM, with visibly appreciable defects present in cells dosed with 50 nM, while more pronounced filament curvature alterations were detectable following treatment with 80 nM. Interestingly, this correlates with the maximum concentration detected in the blood plasma of patients treated with a therapeutic dose of colchicine [42]. Measured filament curvatures in cells treated with 50–80 nM of drug were as high as 2 rad/μm, a value which previous studies associated with MT breakage [43, 44]. Increasing colcemid concentration to 100 nM and 200 nM resulted in less abundant cellular MT filaments which appeared shorter, demonstrating that filaments had become fragmented. To further probe the effects of low colcemid doses < 30 nM, we applied a SOFI time-lapse approach to capture the dynamics of individual filaments with a temporal resolution of 20 s (one SOFI image every 20 s) for up to 9 min. Compared with untreated cells, treatment with as low as 18 nM colcemid caused individual filaments to be significantly less active, both in terms of growth and shrinkage, indicating suppression of MT dynamics. Despite a consensus that hindering filament dynamics is a mechanism for cancer therapy, direct observation and quantification of filament dynamics in living cells remain challenging. The super-resolution assays developed here have revealed new insights into the non-mitotic effects of the MT-interacting drug colcemid, namely suppressed filament dynamics, aberrant filament curvature and filament fragments, that may serve potential therapeutic roles against cancer. This study also establishes a new standard for probing the sub-diffraction landscape of the MT network and its perturbations induced by MT-interacting drugs.

## Results

### Low doses of colcemid cause remodelling of microtubule architecture

**Fig. 1:**
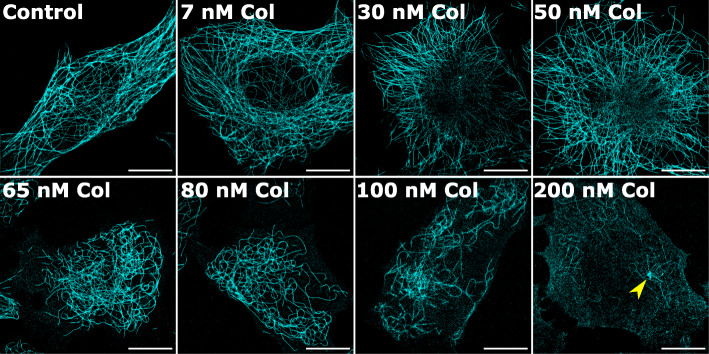
Single-molecule super-resolution imaging (*d*STORM) of MTs in HeLa cells treated with colcemid reveals increasingly aberrant filament curvature. Cells were treated with colcemid at the specified concentrations for 5 h before fixation. Cells were immunolabelled for tubulin with Alexa Fluor 647, imaged and super-resolution images rendered using rapi*d*STORM. Increasingly abnormal curvatures and fragmentation of the MT architecture can be visually detected with significant losses observed at 100–200 nM. The yellow arrow in the 200-nM Col-treated cell indicates a MTOC. Images are representative of each treatment. *N* > 20 cells per condition. Scale bars = 10 μm

*d*STORM was used to detect sub-diffraction effects of colcemid by immunolabelling tubulin in fixed HeLa cells treated with 0–200 nM colcemid for 5 h (Fig. 1). We found 5-h drug incubations to be the shortest period that induced the most variability in MT structure across this concentration range. Protocols for sample preparation and acquisition parameters for *d*STORM of MTs had already been established in our lab using COS-7 cells and applied previously to quantify viral protein-induced MT bundling [37]. In this study, investigating the effects of colcemid, a compound with related colchicine-based synthetics under development for cancer therapy, we employed HeLa cells as a model cancer cell system. Visual inspection of the *d*STORM images of whole-cell MT architecture revealed clear changes to filament shape, abundance and arrangement with increased colcemid concentrations. At 7 nM and 30 nM colcemid, MTs appeared similar to the control, having relatively linear filaments oriented toward the cell’s edge from the centre. At 50 nM colcemid, while filaments were still mostly linear, some filament sections had become visibly more curved; 65 nM and 80 nM colcemid treatments resulted in more pronounced filament curvature throughout the cell. Treatments with 100 nM and 200 nM colcemid resulted in a lower number of filaments that were relatively shorter, consistent with the well-established MT depolymerizing ability of colcemid (and colchicine) [45]. We hypothesized that these short filaments were fragments derived from longer filaments that had broken due to excessive curvature. There was also an increase in “speckled” signals dispersed throughout the cytoplasm that could be from MT filament constituents (*αβ*-tubulin dimers/oligomers). Some cells treated with 200 nM colcemid showed a remaining small, dense structure located adjacent to the nucleus (Additional file 1: Fig. S1). Two-colour *d*STORM with gamma-tubulin, an established marker for microtubule organizing centres (MTOCs) [46], confirmed this to be the identity of these dense structures (Additional file 1: Fig. S2). This is consistent with the current understanding that MT depolymerization is induced from the + end of filaments oriented at the cell periphery [47].

Notably, 80-nM colcemid-treated cells, wherein aberrant filament curvature was detected by visual appraisal, equate to the maximum concentration found in the blood plasma of patients treated with therapeutic doses of colchicine [41]. Blood concentrations above this, and more comparable to the 100–200 nM colcemid treatments that caused gross MT network destabilization, have been associated with lethal doses. In these studies [48, 49], drug concentrations were measured from solution in direct contact with patient cells. Similarly, in our experiments, we inferred colcemid concentration from the final growth medium of HeLa cells.

**Fig. 2:**
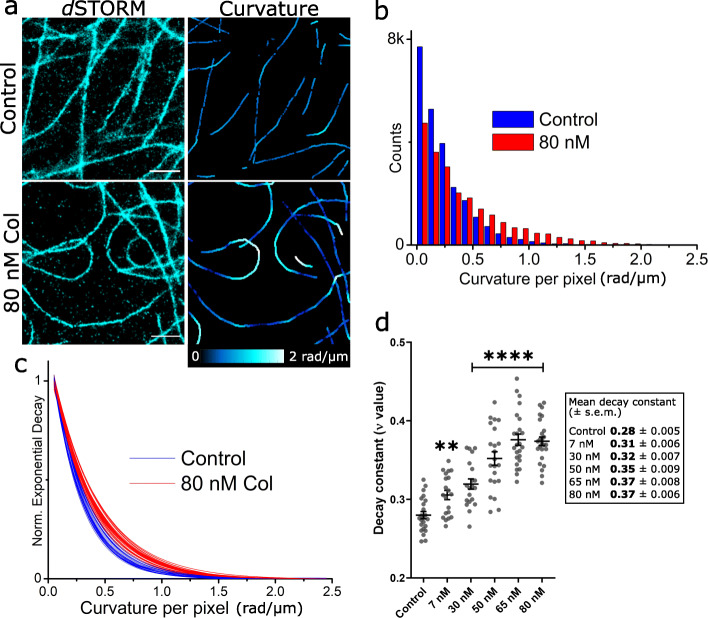
Analysis of filament curvature induced by colcemid. **a** Filaments from *d*STORM images of control and 80-nM colcemid-treated HeLa cells (left). Curvature analysis using SIFNE and coloured at each traced pixel for curvature between 0 and 2 rad/μm. SIFNE curvature output has been dilated by 10 pixels (using ImageJ) to enable visualization. Original SIFNE output shown in Additional file 1: Fig. S3. Scale bars = 1 μm. **b** Histogram of curvature data (curvature at each pixel) from a control cell and 80-nM colcemid cell with bins every 0.1 rad/μm. **c** Normalized exponential decays from histograms of control (*N* = 23) and 80-nM colcemid-treated cells (*N* = 25). **d** *ν* value of each cell analysed with SIFNE (*N* = 135 cells, 50.4 mm total filament length) with mean and standard error of means. All *ν* values were derived from exponential fits with adjusted *R*^2^ > 0.994. Parametric *t*-tests for each colcemid concentration against the control reveal a significant difference with 7 nM colcemid (**p = 0.0014) and each higher colcemid concentration 30–80 nM (****p < 0.0001). Data for **d** are provided in Additional file 3: Sheet 1

To quantify filament curvature from the *d*STORM images, we used SIFNE (SMLM image filament network extractor) [50] to calculate the extent of curvature at each pixel along traced filaments (Fig. 2). Each curvature value was obtained as the reciprocal of the radius of a circle derived from the rate of curvature (Additional file 1: Fig. S3) and determined every 20 nm along a continuous filament. Each cell analysed provided at least 14,000 curvature values from several hundred microns of summed filament length. Curvatures ~ 1 rad/μm related to intermediate filament curves whereas those beyond 1.5 rad/μm were associated with more extensive and aberrant filament curvatures.

Curvature data histograms from each cell were fitted by a single exponential decay function to obtain a decay constant (presented as itsa reciprocal or *ν* value) which provided an empirical measure of the distribution of curvature where a larger *ν* implied a greater proportion of higher curvature values in the histogram and therefore a larger “average curvature” for the MT filaments of that cell. The same analysis was performed on all cells (0–80 nM), and the resulting *ν* values showed a clear trend of MT curvature increasing with colcemid concentration. Cells with the highest proportion of curvatures were those treated with 65 nM and 80 nM of colcemid. With 100 nM and 200 nM colcemid-treated cells, however, we found SIFNE could not properly trace the few filaments present despite their prominence in the *d*STORM images. The high density of speckled signal present in those cells interfered with SIFNE’s tracing algorithm to generate false filaments by joining specks together. These results match the visual inspection of the *d*STORM images where MTs are more curved but still intact at 65 nM and 80 nM and clearly fragmented at higher concentrations.

Overall, *d*STORM paired with SIFNE were employed to characterize both macro and sub-diffraction changes to MT filament structure induced by colcemid. Of the cells analysed for filament curvature, the mean *ν* values increased with increasing colcemid concentrations from 7 to 80 nM compared to the control. This suggested that filaments in the colcemid-treated cells became more perturbed with additional colcemid, initially sustaining some mild curvature with lower doses (7–30 nM) then visually striking aberrant curvatures with higher doses (65–80 nM). Remarkably, even the lowest dose tested was found to have significant effects on MT architecture despite no obvious filament perturbations observed in the corresponding *d*STORM images. The detection of MT structural effects, however mild, suggested that MT dynamics may also be impaired. Therefore, we employed live-cell SOFI to probe MT filament dynamics in response to colcemid.

### Sub-diffraction time-lapse imaging of live-cell microtubules correlates with fixed-cell imaging and reveals suppression of MT dynamics with low colcemid treatment

**Fig. 3:**
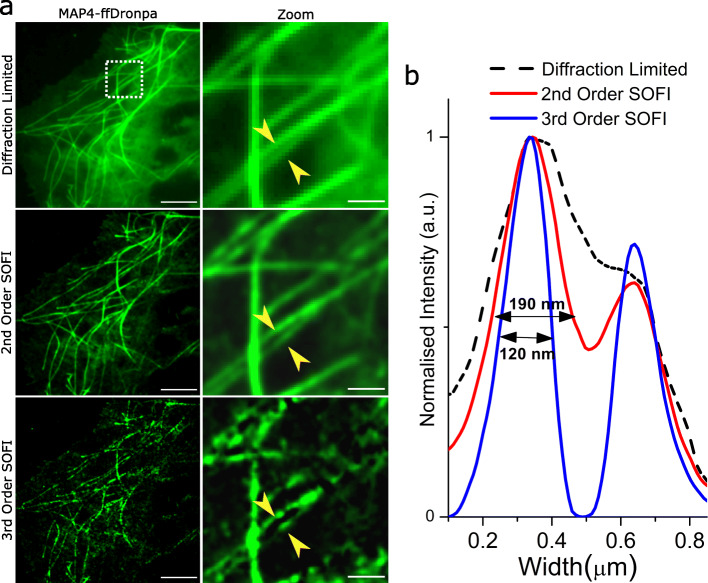
Second-order SOFI produces optimal trade-off between sub-diffraction resolution and image clarity of live-cell MT filaments. **a** Representative diffraction-limited, 2nd- and 3rd-order SOFI images of MTs in a COS-7 cell labelled with MAP4-ffDronpa. Zoomed regions (dotted white box) show the same proximate filaments (yellow arrows) highlighting improved separation in the 2nd- and 3rd-order renderings, but increased loss of structure and clarity in the 3rd-order image. Scale bars = 5 μm (left-hand images) and 1 μm (right-hand images). **b** Intensity cross-sections across the proximate filaments showing improved resolution with 2nd- and 3rd-order SOFI. Values are FWHM of Gaussian fitted to the first peaks of the intensity plots. A Gaussian fitted to the first peak of the diffraction-limited trace (dotted line) resulted in a FWHM of 450 nm, demonstrating the poor image resolution. Data for **b** are provided in Additional file 3: Sheet 2

SOFI is advantageous for time-lapse imaging of live cells because it uses comparatively low excitation powers and can generate images in considerably less measurement time than *d*STORM. To establish acquisition and processing parameters for our SOFI assay, we used COS-7 cells that are a common cell line for optimizing new super-resolution assays [51–53], providing a distinct MT network architecture where individual filaments are readily resolved. As a post-processing super-resolution technique, the degree of SOFI order applied determined the spatial resolution gain achievable. We compared the 2nd and 3rd order for our data, and while the resolution improvement was greater for the 3rd order, we selected the 2nd order for tracing individual filaments, since it gave improved structural clarity of MT filaments in both COS-7 cells (Fig. 3) and HeLa cells (Additional file 1: Fig. S4). Another parameter optimized was the duration of real-time raw data correlated to render a single super-resolution SOFI image (integration time (*I*_time_)). The length of collated raw frames dictated not only image clarity, but also the temporal resolution of SOFI time-lapse movies. Because SOFI required several seconds of acquired data to form one sub-diffraction image, cell movement during this period contributed blurry features to the SOFI image. The flexible motions of MT filaments in live cells could result in over-estimating widths or conceal multiple individual filaments as one wider filament. Although we found *I*_time_ = 10 s was sufficient to generate SOFI images, we used *I*_time_ = 20 s for time-lapse movie generation in order to achieve higher clarity of individual MT filaments throughout the observation period.

**Fig. 4:**
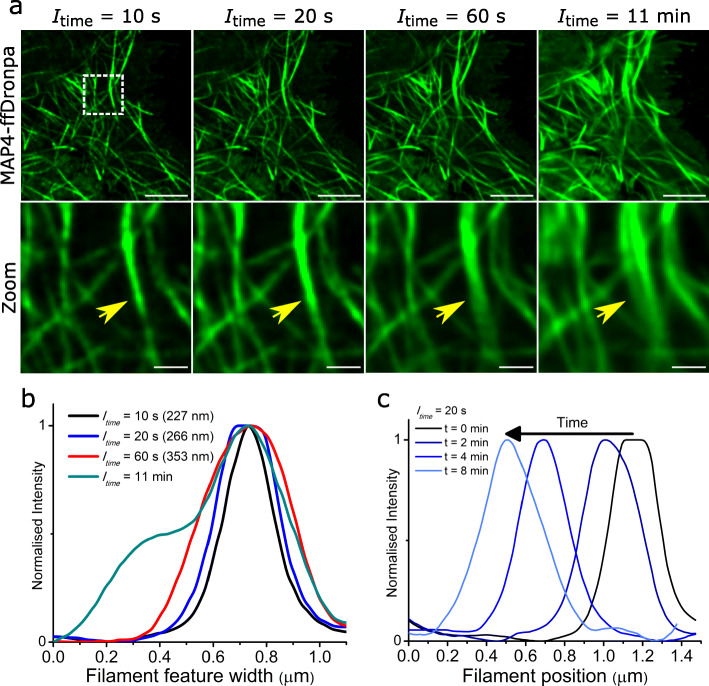
Shorter SOFI integration times reduce smear caused by filament movement while retaining sub-diffraction resolutions. **a** First frames from SOFI time-lapse of live COS-7 MTs labelled with MAP4-ffDronpa and processed using different integration times (*I*_time_) from 10 s to 11 min. Zoom region (dotted white box) from each *I*_time_ image showing the same filament feature indicated by yellow arrow (possibly more than 1 filament) having increasing width with increasing *I*_time_. Scale bars = 5 μm (top row) and 1 μm (bottom row). **b** Intensity profile of filament feature width in **a** with different *I*_time_ and corresponding FWHM values after fitting with a Gaussian. **c** Filament feature width displacement through SOFI time-lapse with *I*_time_ = 20 s at *t* = 0, 2, 4 and 8 min

**Fig. 5:**
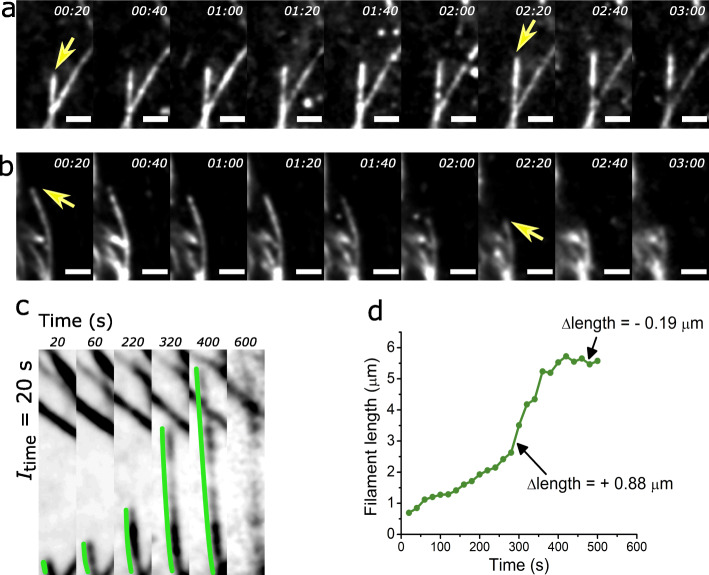
Tracing filament activity from SOFI time-lapse enables sub-diffraction changes in filament length to be quantified. **a**, **b** Consecutive SOFI frames of *I*_time_ = 20 s showing sub-minute dynamics of **a** growing and **b** shrinking MT activity in HeLa cells. Yellow arrows indicate filament of interest. Scale bars = 1 μm. **c** Tracing a growing MT filament from a SOFI time-lapse. Green line measures the length from the reference base to the tip, except in the final frame of *I*_time_ = 20 s where filament becomes unclear. **d** Filament from **c** traced at each frame for 10 min with arrows indicating example measurements for both growth and shrinkage ∆length between successive frames

From SOFI time-lapse experiments, we found filaments could be clearly seen to grow and shrink; however, lateral motions were also observed and caused smearing in the SOFI image (Fig. 4). While these lateral movements were also related to MT dynamicity and function, for this study, we focused on the growing and shrinking of filaments at the tips as a measure of MT dynamics. Filament dynamics can be quantified by measuring growth and shrinking events based on just the filament tip (e.g. using a fluorescent label on MT tip protein EB3 [54]), but because we were able to visualize a whole filament with sub-diffraction resolution, we measured filament growth and shrinkage by tracing several microns from a reference base to the filament tip in each SOFI time-lapse frame (Fig. 5). In this way, live MT activity was measured from SOFI time-lapse movies, and the changes in length (∆length) of individual filaments over time were quantified to elucidate MT dynamics with a 20-s temporal resolution.

**Fig. 6:**
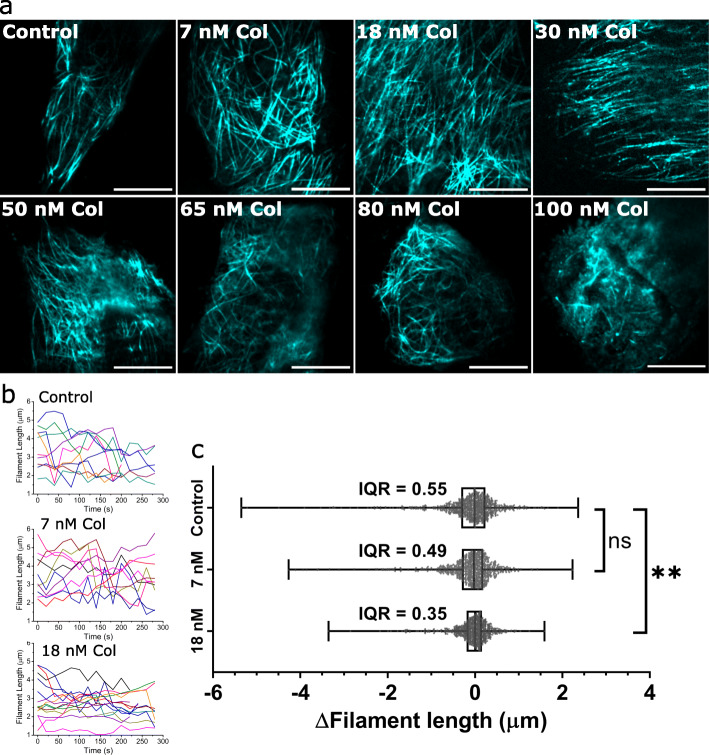
SOFI reveals colcemid causes filament curvature and suppresses filament dynamics in live cells. **a** SOFI of live HeLa MTs labelled with MAP4-ffDronpa, imaged after 5 h treatment with the specific concentration of colcemid. Live cell MTs imaged with SOFI show a similar trend to fixed-cell MTs imaged with *d*STORM. Aberrant filament curvatures were observed in 65 nM and 80 nM colcemid-treated cells with some degree of fragmentation evident in 100-nM colcemid-treated cells. Each cell is representative of each condition imaged with SOFI (SI Fig. 5). Scale bars = 5 μm. **b** Overlay of 10 representative filament traces from SOFI time-lapses of control, 7-nM and 18-nM colcemid-treated cells. Representative SOFI time-lapse movies of each condition are shown in Additional file 2. **c** Length values were extracted between each consecutive frame and used to determine the given ∆length for each pair of frames. Distributions of ∆length values obtained from 3 independent assays, *N* = 12 cells, > 96 filaments and > 770 ∆length events for each condition. Scatter plots of data overlaid with median, interquartile range (IQR, box) and full range (whiskers). The Kolmogorov-Smirnov test was used to determine the significant difference between control and 18 nM colcemid treatment (**p = 0.0011). Data for **c** are provided in Additional file 3: Sheet 3

Static SOFI images of colcemid-treated HeLa cells showed similarities to those imaged with *d*STORM. Cells treated with 7–50 nM colcemid had filaments of similar appearance to the control cells with mostly linear filaments extending outward from the centre (Fig. 6). At 65 nM and 80 nM colcemid, there was more curvature present with more filaments curved inward away from the cell edge. In 100 nM colcemid-treated samples, a small number of abnormally short filaments could be detected. SIFNE, however, could not accurately trace individual filaments for curvature analysis given the poorer resolution of MT filaments in the 2nd-order SOFI images compared to *d*STORM. Overall, the trend from visual inspection of live cells matched that observed from *d*STORM and therefore demonstrated that abnormal curvatures quantified from *d*STORM accurately recapitulated the MT architecture in drugged living cells. We also hypothesized that the observable increase in unstructured “non-specific” fluorescence signal seen in ≥ 65 nM colcemid-treated cells corresponded to the well-resolved speckled signals detected in the *d*STORM images. This was explained by the increase in MT breakage and disassembly resulting in higher concentrations of dimeric/oligomeric tubulin. While these individual species could be resolved as speckles in the *d*STORM images of fixed cells, the poorer resolution achieved with the 2nd-order SOFI, combined with the dynamic movement of these species within live cells, resulted in a higher intensity of unstructured blur. The effects of these dynamics combined with out-of-plane fluorescence also manifested as the uneven filament signal observed in cells imaged with SOFI (Additional file 1: Fig. S5). Because correlation was based on the quality of fluctuations, weaker fluctuations further away from the focal plane (higher planes) yielded dimmer SOFI-calculated pixels meaning those MTs perfectly in focus presented more brightly than those slightly out of focus.

For SOFI time-lapse assays, we applied 2nd-order correlation to achieve both sub-diffraction resolution and high image clarity of individual live-cell MT filaments. We also used *I*_time_ = 20 s for the best temporal resolution that retained the clarity of filaments while minimizing the amount of smearing caused by lateral filament movements. By tracing individual filaments over several minutes, we were able to quantify instants of both growth and shrinkage. These optimized SOFI parameters were used to interrogate low colcemid concentrations (7 nM and 18 nM) where although no visible filament deformations were observed in either the *d*STORM or SOFI images, significant increases to filament curvature hinted that MT dynamics were also affected. Because MT filaments behave differently depending on their position in the cell [55], sampling of filaments for dynamics imaging and tracing was consistent, i.e. all measured filaments were from the edges of cells with the presumption that colcemid effects would be most prominent there. Traces of 10 representative filaments from each condition show 18 nM colcemid-treated filaments had relatively smoother traces than the control traces, indicative of slower dynamics. From each filament trace, the change in filament length between SOFI time-lapse frames (∆length), effectively the change in length over 20 s of real time, was measured. Multiple measurement points (14 ∆length values for a 5-min time-lapse) from a single filament were compiled and visualized as the total distribution of ∆length for each condition. This analysis revealed that the overall distribution of dynamics between the control and 18 nM colcemid-treated cells was significantly different with the colcemid treatment causing a loss of overall dynamic movement due to both growth and shrinkage occurring more slowly. These results using super-resolution time-lapse imaging demonstrated the loss of MT dynamics with low concentrations of colcemid previously uncharacterized in situ.

## Discussion

Generating super-resolution images and movies allowed novel quantification of the observed responses of MTs in the presence of colcemid. Visual inspection of *d*STORM images of MTs clearly presented a trend of increasing filament curvature with increasing colcemid doses. The application of SIFNE to measure the filament curvature was successful at evaluating the visual trend, owing to the clarity of filaments in the *d*STORM images. In order for SIFNE to trace filaments reliably, *d*STORM images must contain sufficiently high localization density, a condition achieved with sufficiently high fluorophore labelling density and number of acquired SM blinking frames. Insufficient labelling would have resulted in localization gaps, causing filaments to appear discontinuous and become less likely to be properly traced by SIFNE. For the curvature analysis, shorter filaments and speckled features (probably free tubulin units) under 200 nm in length were excluded because with SIFNE’s default settings, these shorter features in close proximity could be joined together into false filaments. While this is a useful feature in SIFNE to reconcile for low localization densities, it is not a substitute for proper fluorophore labelling during sample preparation. Also, this exclusion of shorter features would not have altered the increasing curvature trend quantified since most curved filaments of interest were of substantial length (> 1 μm). Although SIFNE provides several other adjustable parameters, mostly default settings were reliable to trace and measure MT filaments from the high-quality *d*STORM images used. Besides SIFNE, there are other analytical tools to trace and measure super-resolved MT filament networks, such as LineProfiler [56] that can quantify MT widths throughout an entire imaged MT network. As a preliminary demonstration, we applied LineProfiler to quantify MT bundling induced by the anticancer drug paclitaxel in HeLa cells imaged with *d*STORM (Additional file 1: Fig. S6). Since the antibodies used to fluorescently label 25-nm-wide MT filaments contribute to filament width, and together with the localization precision of *d*STORM, individual MT filaments captured with *d*STORM are typically 60–80 nm wide [6, 37]. MT bundles in paclitaxel-treated cells were observed to reach sizes as large as 300 nm significantly more than filaments in untreated cells. The ability to resolve individual filaments from within MT networks provides a clearer distinction of sub-diffraction anomalies and improves the quality of filament measurements over conventional imaging techniques. Advances to super-resolution imaging since its advent in the early 2000s have evolved techniques such as *d*STORM and SOFI that are readily implemented in most labs with a standard widefield fluorescence microscope. Relative to the discoveries and early characterization of MT-interacting compounds, these modern strategies for direct observation into fixed and live cells provide improved visualization and analysis of subcellular features, permitting revised interpretations of drug mechanisms. Our work interrogating colcemid-induced MT dysfunction has revealed previously uncharacterized subcellular effects and validates the application of super-resolution to study small-molecule drug compounds. The near 20-nm resolution of *d*STORM enabled analysis of aberrant filament curvature, an effect yet to be associated with colcemid or MT-interacting drugs in general. Our extension of SOFI for time-lapse imaging of live cells, while currently achieving only a slight resolution gain over conventional live-cell imaging, possesses undisclosed potential for further improvements in both spatial and temporal resolution to match the scale and speed of MT filament dynamics. Together, these assays contributed to the understanding of subcellular mechanisms of colcemid and can be further applied to image drug-induced dysfunction in other cellular components such as mitochondria and actin.

**Fig. 7:**
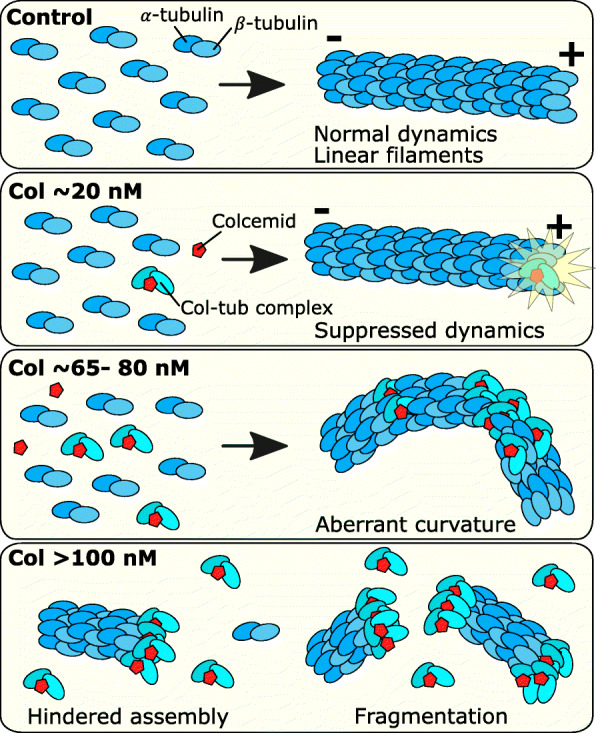
Microtubule dysfunctions associated with different doses of colcemid MT filaments are polymers of *α*- and *β*-tubulin subunits that assemble through dynamic processes. In non-polarized cells, filaments grow toward a positive end and form relatively linear filaments that extend outward from the cell centre toward the cell membrane. With low levels of colcemid ~ 20 nM, MT dynamics are suppressed, and filaments grow and shrink at a slower rate. Increased levels of colcemid ~ 65–80 nM produce more tubulin-colcemid complexes that distort normal tubulin-dimer subunit configurations, resulting in aberrant filament curvatures when these complexes become incorporated. Higher levels of colcemid > 100 nM result in short filaments (filament fragments), either due to curvature strains that result in breakage or the inability for filaments to form properly due to an excess of tubulin-colcemid complexes

The binding of colcemid at the colchicine binding site between *α*- and *β*-tubulin monomers distorts the protein configuration of tubulin dimer subunits [57]. We propose this resulting misshaped *αβ*-tubulin-colcemid complex interferes with the normal end-to-end assembly of typically linear MT filaments, introducing a kink into the filament and subunits become oriented at various angles to one another. With enough of these, filament curvatures would become more apparent (Fig. 7). This is consistent with the increasing frequency and extent of curvatures observed with increasing colcemid concentrations, starting from as low as 50 nM, followed by the appearance of curvatures beyond 2 rad/μm at 65 nM and 80 nM. At 100 nM and 200 nM, some curved MTs were observed together with shorter filament fragments, suggesting that filament breakage may occur as a result of excessive structural strain at these highly curved sites. Also, since these higher drug concentrations result in a larger proportion of drug-affected tubulin in cells, MT filaments may find it more difficult to sustain filament assembly. Therefore, subunits would be less likely to be incorporated into filaments and remain as free cytoplasmic constituents; this idea is supported by our observation using *d*STORM of speckled features in cells after these relatively higher doses of colcemid. Inducing MT filament fragments, either through curvature-derived breakage or inhibition of tubulin assembly, or a combination of both, reflects a toxic outcome because most of the MT network is absent.

Colchicine (and colcemid) binds free tubulin subunits [11] to form tubulin-colchicine complexes that hinder further filament growth by limiting tubulin polymerization when they are incorporated into growing MT filaments [58]. From SOFI time-lapse movies (Additional file 2), we observed this effect where 18 nM colcemid suppressed filament growth and interestingly also reduced the events of filament shrinkage, implying increased filament stability and/or slowed rate of disassembly. These MT perturbations would likely have wide-ranging consequences for various vital MT functions including intracellular transport, cellular mobility and tissue structure. It has been previously shown that a similar range of colchicine (12 nM and 25 nM) significantly reduced the ability of human cells to migrate through 8-μm plastic pores [59]. The lack of aberrant filament curvatures observed up to 30 nM suggests MT-dependent intracellular transport may still have remained viable. Measuring the dynamics of motor proteins could reveal if trafficking/signalling is similarly suppressed by these levels of MT-interacting drugs. Additionally, reduced filament dynamics would affect the genetic segregation during mitosis. Direct imaging of MT alterations both outside and during mitosis would provide a holistic perspective of drug mechanisms at the subcellular scale and could be beneficial in understanding their contributing roles to elicit therapeutic outcomes against cancer and other diseases.

## Conclusion

In conclusion, we have applied complementary super-resolution imaging methodologies to study the impact of colcemid on the dynamics and structures of MTs. We found that increasing colcemid concentration correlated with distinct MT effects in HeLa cells where the transition between each effect was sensitive to concentration increments of several tens of nanomoles. The increasing severity of effects from suppressed MT dynamics at 18 nM to aberrant MT curvatures at 80 nM to substantial loss of the MT network at 100 nM suggests filament curvature could be a visually distinctive marker for impending toxicity of colcemid and possibly other colchicine derivatives. The onset of these MT perturbations after only a short exposure to the drug (5 h) supports the notion that traditional “antimitotics” affect MT function by mechanisms distinct from effects on mitosis. Our studies show that subtle drug effects could be of high importance in understanding the therapeutic value and highlight the emerging potential of super-resolution light microscopies to reveal and characterize the impact of small molecules on complex cellular structures.

## Methods

### Cell culture

HeLa (human epithelial adenocarcinoma, ATCC CCL-2) and COS-7 (African green monkey kidney fibroblast-like, ATCC CRL1651) cells were cultured in Dulbecco’s modified Eagle’s medium (DMEM – high glucose) supplemented with 10% foetal bovine serum and 1% penicillin-streptomycin and incubated at 37 °C with 5% CO_2_. Cell stocks were passaged twice a week to maintain 40–90% confluence in 25-cm^2^ flasks. For live-cell imaging, cells were seeded onto high precision coverglass (Marienfeld 18 mm diameter #1.5H coverglasses, cat #0117580) and grown to 60% confluence before transfection. Seeded cells were transfected using Fugene HD Transfection Kit according to the manufacturer’s instructions (Promega). For each chamber, transfection media comprised 500 ng of DNA plasmid (pMAP4-N1-ffDronpa) and 2 μL Fugene reagent in 50 μL DMEM. Cells were incubated in transfection media for at least 18 h. For drug assays, colcemid (Roche cat #10295892001) was added to cell growth media to yield final concentrations ranging from 7 to 200 nM 5 h before fixation and immunostaining, based on a previously optimized protocol [60]. Typically, colcemid ranging from 100 to 300 nM (40–100 ng/ml) is used in laboratory settings to induce mitotic arrest for synchronizing cell cultures or for chromosome spreading protocols [39]. Similarly, paclitaxel was used at a final concentration of 1 μM for 5 h before fixation and immunolabelling.

### *d*STORM sample preparation

For *d*STORM imaging, untransfected Hela cells were cultured and treated with colcemid as above before being permeabilized in 0.25% Triton X-100, 0.3% glutaraldehyde (Alfa Aesar cat #A17876) in cytoskeletal buffer (CB: 10 mM MES pH 6.1, 150 mM NaCl, 5 mM EGTA, 5 mM MgCl_2_, 5 mM glucose) at 37 °C for 30 s, then fixed in 2% glutaraldehyde in CB for 10 min at 37 °C. Fixed cells were washed in phosphate-buffered saline (PBS) twice for 5 min, then quenched in 0.1% NaBH_4_ in PBS for 7 min at R.T, then washed twice in PBS for 5 min. Cells were blocked in 5% bovine serum albumin in PBS for at least 30 min at R.T. before immunostaining with rabbit anti-*α*-tubulin (Abcam ab18251, 1:500 in 5% BSA/PBS, 1 h, R.T) then anti-rabbit Alexa Fluor 647 conjugate (ThermoFisher cat #A-21246, 1:200 in 5% BSA/PBS, 45 min, R.T). Following each antibody stain, cells were washed in 0.1% Tween-20 in PBS twice for 5 min. For two-colour *d*STORM of beta and gamma tubulin, antibodies for both targets were added simultaneously, i.e. primary antibodies diluted in 5% BSA/PBS (mouse anti-beta-tubulin 1:100, Sigma T8328 and rabbit anti-gamma-tubulin 1:1000, Abcam ab11317), then secondary antibodies diluted in 5% BSA/PBS (anti-mouse Alexa Fluor 647 1:200, ThermoFisher cat #A-21237 and anti-rabbit Alexa Fluor 532 1:200, Thermo cat #A-11009). Cells were then post-fixed in 3.7% formaldehyde for 5 min at R.T. A switching buffer of 100 mM mercaptoethylamine (MEA) in PBS made to pH 8.2 (adjusted with KOH) was added to cells for *d*STORM imaging.

### Single-molecule super-resolution imaging

Imaging was performed on a home-built single-molecule super-resolution widefield microscope as previously described [61]. Briefly, we used an Olympus IX81 inverted fluorescence microscope frame fitted with a TIRF ×100 mag 1.49 NA oil objective, Oxxius 638-nm, Dragon 532-nm and Toptica 488-nm laser diodes, and Andor iXon EM-CCD detector. Acquisition parameters were controlled using Micromanager. For *d*STORM, cells were imaged in a switching buffer of 100 mM mercaptoethylamine (MEA) at pH 8.5 in PBS. The 638-nm laser was used at full power (150 mW resulting in 3–5 kW/cm^2^) to induce photoswitching of Alexa Fluor 647 for resolved single-molecule emissions (observed as “blinking” events) that were acquired using Micromanager [62] (20 ms exposure, 100 gain, > 10,000 frames). Imaging was performed in quasi-TIRF mode that captured emissions from a limited axial range of a few micrometres above the coverslip. Acquired frames were analysed in rapi*d*STORM [63] using input pixel size 100 nm and point spread function full width half maximum (PSF FWHM) of 360 nm to localize each single-molecule emission. A reconstructed coordinate map of all localizations (over 1 million per image) produced the 2D super-resolved *d*STORM image.

### Filament curvature analysis

The contrast of *d*STORM images was enhanced with a Gaussian blur (ImageJ [64], sigma (radius) = 1) to enable better MT tracing from SIFNE [50]. For measuring filament curvature, MT images were processed through SIFNE with default parameters except “Max Curvature” which was set to 3 rad/μm. This was to accommodate detection of more extreme curvatures and curvatures occurring along the axial plane (curvature along the *z*-axis would be more pronounced when visualized from orthogonal 2D perspectives). At the last step of the SIFNE analysis to avoid potentially fragment filaments, a minimum length of 200 nm was set for inclusion in the analysis. The curvature of each pixel traced by SIFNE (at least 14,000 per cell) was compiled into a histogram (0.1 rad/μm bins) and fitted with a single exponential decay function:

$$ A={A}_0{\exp}^{-\theta /v} $$

where *A* is the proportion of curvature events with a given curvature *θ*, *A*_0_ is the initial amplitude at *θ* = 0, and *ν* is the reciprocal of the first-order decay rate constant. Cells from 2 independent assays of each colcemid condition were compiled to determine the mean decay constant. Unpaired parametric *t*-test with Welch’s correction was performed on compiled *ν* values for each colcemid concentration against the control. A significant difference was found with 7 nM colcemid treatment (**p = 0.0014) and each higher colcemid treatment 30–80 nM (****p < 0.0001).

### Filament width analysis

*d*STORM images of MTs in control and paclitaxel-treated HeLa cells were obtained using the same procedure as described previously. For each cell analysed (5 cells from each control and drugged), four 10 μm × 10 μm areas were processed through the LineProfiler interface using the “Microtubule” setting and with default parameters. The output yielded average filament widths from ~ 1–3 μm of filament length, with each cell providing at least 80 average width values. Control cells that presented with an overall higher density of thinner filaments resulted in more average width values. These average filament feature widths were plotted for each condition, control and drugged, to determine the mean ± standard error of the mean (control 86 ± 1 nm, 1 μM TAX 111 ± 2 nm). A significant difference was determined using a parametric unpaired *t*-test (****p < 0.0001), indicating paclitaxel-induced MT bundling.

### SOFI acquisition

SOFI was performed on the same setup as *d*STORM experiments. HeLa cells were grown on coverglasses and labelled by transfection with a DNA plasmid for transient expression of ffDronpa conjugated to microtubule associating protein 4 (MAP4). Just before imaging, transfected cells were rinsed with warm PBS then mounted in a custom-built chamber and filled with warm PBS. Upon excitation with continuous 488-nm laser at ~ 50 mW/cm^2^ (Toptica, total output = 2 mW), ffDronpa photoswitched at rates suitable for SOFI analysis. ffDronpa was also stably fluorescent at even lower power (~ 5 mW/cm^2^, 0.2 mW), however, was found to be insufficient for photoswitching. Ideal raw data for SOFI is a dense coverage of fluctuations across the labelled structure. As such, the general shape of MT filaments is clearly visible during SOFI acquisition, unlike for *d*STORM where only single molecule emissions are observed. Relevant to both techniques is that the quality of raw data determines the quality of super-resolution images. During acquisition, cells were exposed to cumulative laser exposure for no longer than 10 min. Live-cell SOFI raw data acquisition was performed using Micromanager with 100 gain at 20 Hz and saved as .tif movie stacks. Between 2000 and 6000 frames were collected from each cell depending on signal quality over time.

### SOFI processing

Acquired frames were imported into the Localizer package [65] in Igor Pro 7. Under the SOFI tab, the following parameters were applied for static SOFI images: order = 2, pixel combos = more, also average image = checked, and frames 0–399. Executing the analysis produced (i) the average image combining all acquired frames, representative of a diffraction-limited fluorescence image, and (ii) the correlated SOFI image. Finally, a Richardson-Lucy deconvolution was applied; standard deviation of the PSF = 1.6 pixels, number of iterations = 2. The contrast of the image was further modified by selecting Macros>Append colour scale sliders and adjusting the upper and lower limits of the final SOFI image. The processing sequence is identical to create a SOFI time-lapse with the exception of selecting “Make movie” and selecting the number of acquired frames to be correlated into each SOFI frame. Acquired data of several thousand frames were correlated every 400 frames (accounts for 20 s of real-time data) to produce a continuous SOFI time-lapse up to 5 min. Integration time (*I*_time_) describes the duration of real-time data used to generate one SOFI image or one frame in a SOFI time-lapse (Fig. 4). The Localizer interface enables control of *I*_time_ by selecting the number of acquired frames per SOFI image: 200 frames = 10 s, 400 frames = 20 s, 1200 frames = 1 min, 13,000 frames = 11 min. Lower integration times produced more fluid SOFI time-lapses of MT dynamics (higher temporal resolution) but lacked improved spatial resolutions and image clarity of higher integration times.

### SOFI orders for improving resolution and image clarity

To test the correlation parameters, the same data were processed using the 2nd- and 3rd-order SOFI correlations (Fig. 3 and Additional file 1: Fig. S4). Compared to the diffraction-limited fluorescence image, both SOFI correlation orders enhanced the contrast and clarity of individual MT filaments. In the zoomed in regions, it becomes clear that the number of pixels increases (and pixel size decreases) through the formation of “virtual pixels” inherent to the SOFI process [66]. Using FWHM of filament intensity cross-sections, widths were found to be in the range of 120–190 nm, which is about a factor of 2 to 3 improvement over the measurement from the original diffraction-limited image (unresolved ~ 450 nm in Fig. 3) as expected for the 2nd- and 3rd-order SOFI analysis respectively. Although this is not as good a resolution gain as can be achieved using *d*STORM, SOFI provides more biological relevance with live-cell imaging. Comparing between SOFI orders, we found the 3rd order yielded a better distinction of two adjacent filaments than the 2nd order. However, a significant number of filaments correlated by the 3rd- order were discontinuous, and the overall MT network was less visible. Despite the additional resolution improvement from the 3rd order, we used the 2nd order SOFI for all subsequent analysis, given its consistency in retaining the integrity of whole filaments throughout the entire image. Subsequent SOFI for static images and time-lapses were performed using consistent acquisition parameters (20 Hz acquisition framerate for at least 20 s) and processed with 2nd-order SOFI correlations.

### Optimizing *I*_time_ for SOFI time-lapse

To achieve clear MT filaments throughout the time-lapse, we required a minimum *I*_time_ of 20 s (400 acquired frames acquired at 20 Hz) per SOFI frame which corresponds to 20 s temporal resolution. We tested different integration times (*I*_time_ = 10 s, 20 s, 1 min, 11 min) on acquired data of living MTs to make SOFI time-lapses with different temporal resolutions (Fig. 4). The movement of MTs throughout the acquisition, when integrated, produced smearing artefacts with longer *I*_time_ that added apparent size to structures. A measured filament feature (possibly more than one filament) increased in width from 230 nm up to > 500 nm by increasing *I*_time_ from 10 s to 11 min due to its lateral movement during acquisition. Using *I*_time_ = 20 s, we could track its displacement across several hundred nanometers after 2, 4 and 8 min of real time with a similar width retained throughout. Though filaments could be rendered using *I*_time_ = 10 s for better temporal resolution, each SOFI frame had relatively less signal, and it was more difficult to resolve continuous structures. This was especially apparent after ~ 9 min of continuous imaging where ffDronpa signal was reduced due to photobleaching. By using *I*_time_ = 20 s for all SOFI time-lapse experiments (and SOFI images in previous images), we compromised temporal resolution for longer observation times of MT dynamics with better filament contrast and clarity.

### Tracing live MT filaments

Filaments at the cell edge were selected for analysing dynamics because colcemid-induced depolymerization occurs from filament (+) ends, and so we hypothesized that initial interruption to MT dynamics would manifest most prominently at the edges. Additionally, cells are typically thinnest at the edges compared to the centre, meaning imaging at these areas minimized the amount of out-of-focus fluorescence, improving the quality of the raw and rendered SOFI images. We used the segmented line tool in ImageJ to trace each filament by selecting a reference base point and measured to the tip at each SOFI frame throughout the SOFI time-lapse (Fig. 5). Each length was plotted as a function of time to form a filament trace. Because photobleaching of RSFPs becomes more apparent in the later acquisition frames labelled structures in later SOFI time-lapse frames may not be as clear. The example in Fig. 5 is the filament (imaged with *I*_time_ = 20 s) at *t* = 600 s that is barely visible and could not be traced because of the degrading RSFP signal. However, at least 5 min of traceable filaments was obtainable from each cell and up to 9 min in some instances.

### Measuring drug-affected microtubule dynamics

We performed triplicate assays of each colcemid treatment (0 nM, 7 nM, 18 nM) for measuring microtubule dynamics using SOFI. We sampled at least 8 filaments from each cell (4 cells per assay) using SOFI time-lapse movies. In each SOFI frame, filaments were measured from a reference base to the tip using the segmented line tool in ImageJ. Measurements were used to plot the change in length (∆length) of each filament every 20 s. All ∆length (+ve and –ve) were compiled for each drug condition as a scatter plot and overlaid with a box-and-whisker plot of the interquartile range (IQR) and full range (whiskers). ∆length values when combined were not normally distributed, failing normality tests (D’Agostino-Pearson test and Shapiro-Wilk test in GraphPad Prism 8). This is consistent with the idea that accumulated MT growth and shrinkage rates would not have normal distributions given the presence of other proteins that regulate MT dynamics [67] and that the rate of MT disassembly is typically faster than assembly. Given the non-normal distribution, we used the IQR to describe the spread of ∆length values. Compared to the control, we observed a narrower distribution from the 18 nM colcemid data, indicative of suppressed MT dynamicity, i.e. the lengths of traced filaments increased and decreased less or at slower rates during the periods of observation. The reduced IQR shows overall hindered filament activity by about 11% and 36% with 7 nM and 18 nM colcemid treatment respectively. Applying the Kolmogorov-Smirnov test, we found 18 nM colcemid induced a significant difference (**p = 0.0011) to the distribution of ∆length values compared to the control.

## Supplementary Information

**Additional file 1: Fig. S1.** *d*STORM images of HeLa cells treated with colcemid, labelled for microtubules. **Fig. S2.** Two-colour *d*STORM images of beta-tubulin and gamma-tubulin in colcemid-treated HeLa cells. **Fig. S3.** Graphic summary of microtubule filament curvature analysis. **Fig. S4.** 2^nd^ and 3^rd^ order SOFI images and width analysis of HeLa microtubule filaments. **Fig. S5.** SOFI images of HeLa cells treated with colcemid, labelled for microtubules. **Fig. S6.** *d*STORM of HeLa cell treated with paclitaxel and microtubule width analysis.

**Additional file 2: Supplementary Movie 1.** 2^nd^ order SOFI time-lapse movie of HeLa cells treated with 7 nM and 18 nM colcemid.

**Additional file 3: Supplementary Data 1. Sheet 1.** Datapoints for Fig. 2d. **Sheet 2.** Datapoints for Fig. 3b. **Sheet 3.** Datapoints for Fig. 6c. **Sheet 4.** Datapoints for Fig. S6e.

## Data Availability

All data generated or analysed during the study are included in the published article and its supplementary information files. Super-resolution images generated for this study can be found online at doi:10.26180/16570356.
