# Supplementary material for: Nanoscale characterization of drug-induced microtubule filament dysfunction using super-resolution microscopy
Source: BMC Biol. 2021 Dec 11;19:260. doi: 10.1186/s12915-021-01164-4 (PMC8665533; doi:10.1186/s12915-021-01164-4)
Supplement: Supplementary file 1 — Additional file 1: Fig. S1. dSTORM images of HeLa cells treated with colcemid, labelled for microtubules. Fig. S2. Two-colour dSTORM images of beta-tubulin and gamma-tubulin in colcemid-treated HeLa cells. Fig. S3. Graphic summary of microtubule filament curvature analysis. Fig. S4. 2nd and 3rd order SOFI images and width analysis of HeLa microtubule filaments. Fig. S5. SOFI images of HeLa cells treated with colcemid, labelled for microtubules. Fig. S6. dSTORM of HeLa cell treated with paclitaxel and microtubule width analysis. [file 12915_2021_1164_MOESM1_ESM.docx]

Additional file 1

“Nanoscale Characterization of Drug-Induced Microtubule Filament Dysfunction using Super-Resolution Microscopy”

## Ashley M Rozario, Sam Duwe´, Cade Elliott, Riley B Hargreaves, Gregory W Moseley, Peter Dedecker, Donna R Whelan, and Toby D M Bell

## Supplementary Figures S1 – S6


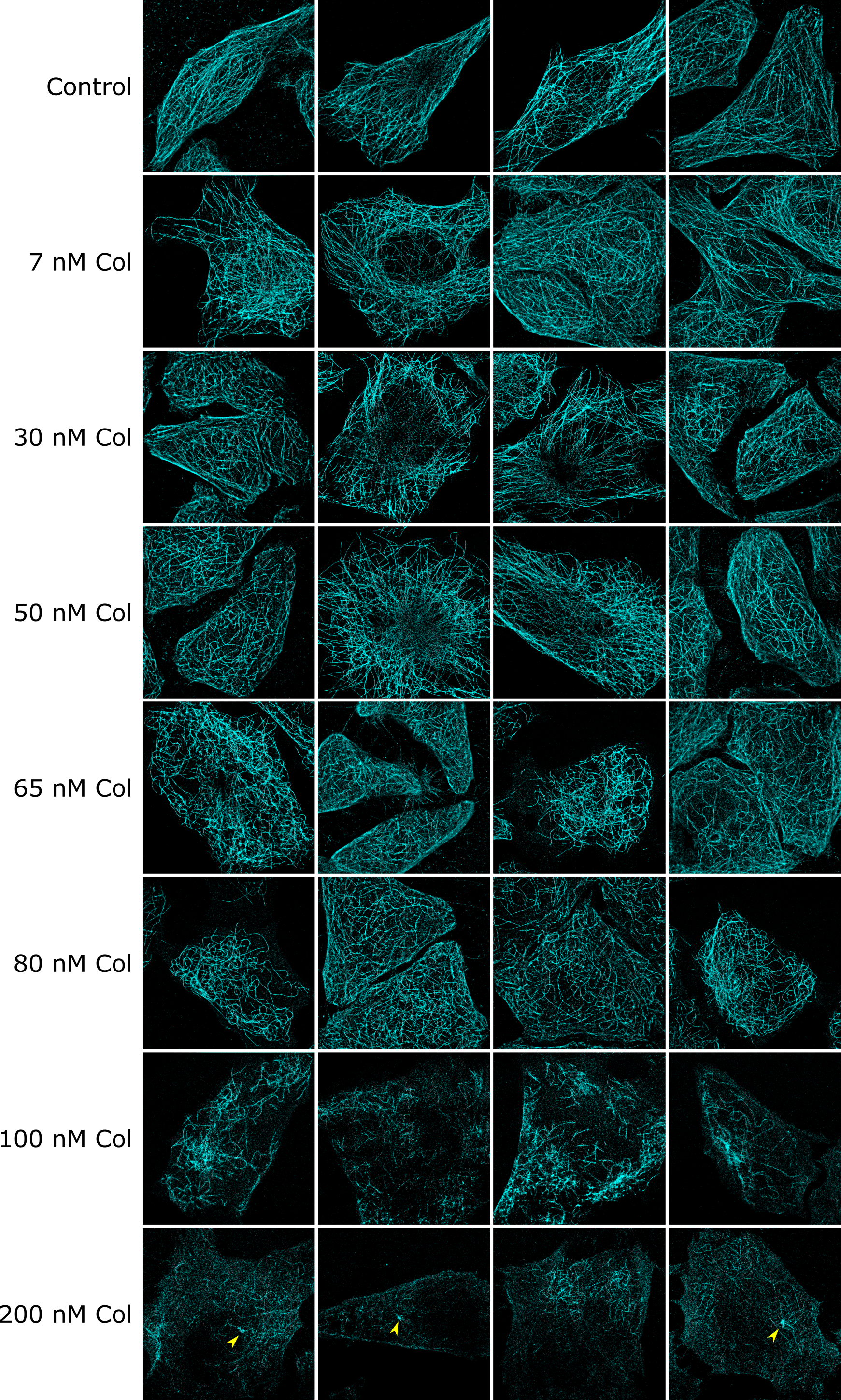


**Fig S1.** HeLa cells from 2 independent assays treated with colcemid and imaged with *d*STORM. Each frame is 40 µm X 40 µm. Yellow arrows in final row indicate possible MTOCs visualized after 200 nM colcemid treatment.

**Fig S2.** Two-colour *d*STORM of beta tubulin (cyan) and gamma tubulin (yellow), labelled with Alexa Fluor 647 and Alexa Fluor 532 respectively, in fixed HeLa cells (top and bottom panels) treated with 200 nM colcemid for 5 hours. Consistent with previous experiments, 200 nM colcemid induced short and few MT filaments. The close proximity of densely labelled structures in both channels (white dotted box) indicates these features are MTOCs. Scale bars = 10 µm (whole cell images) and 1 µm (zoomed images).


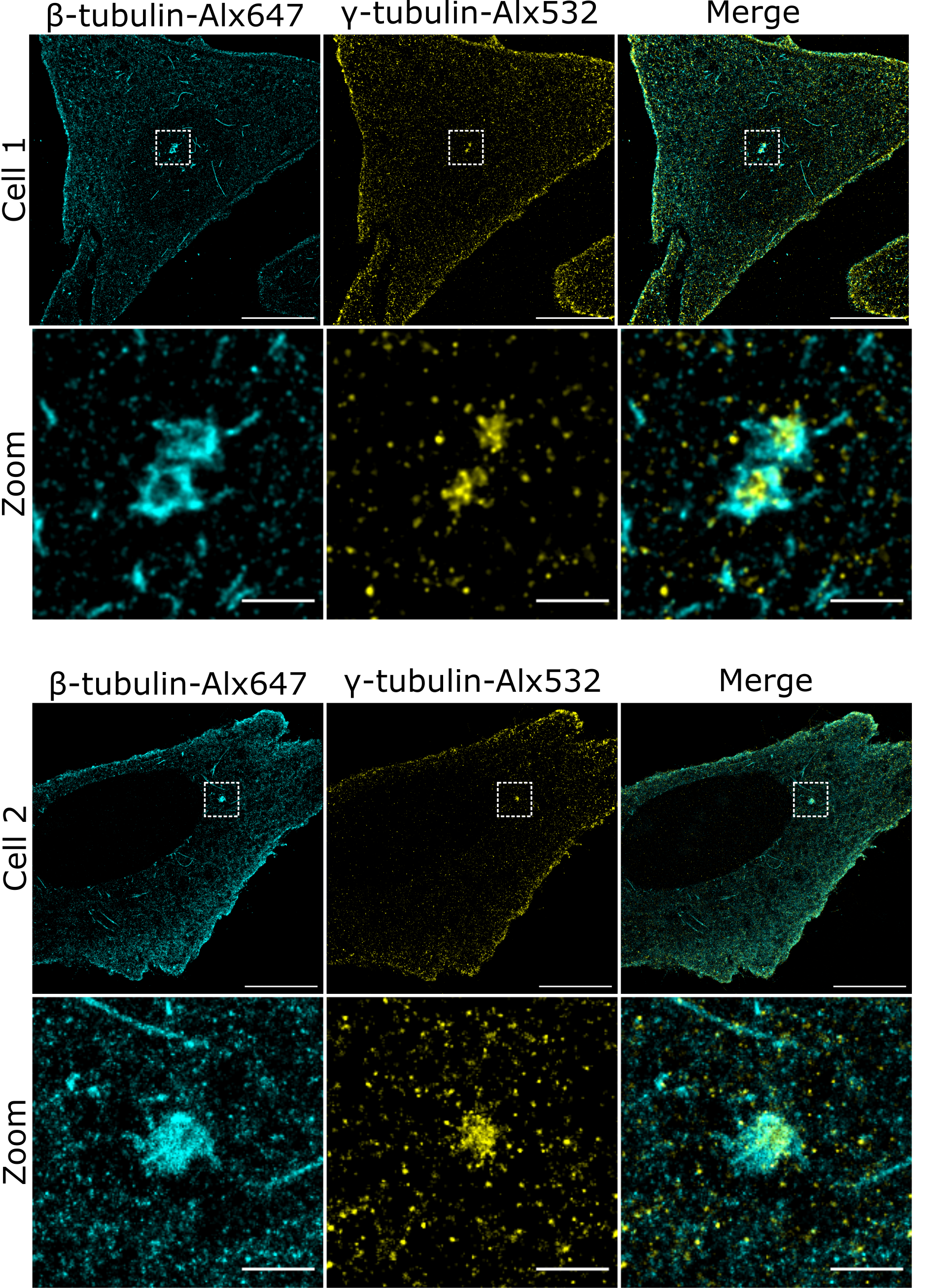

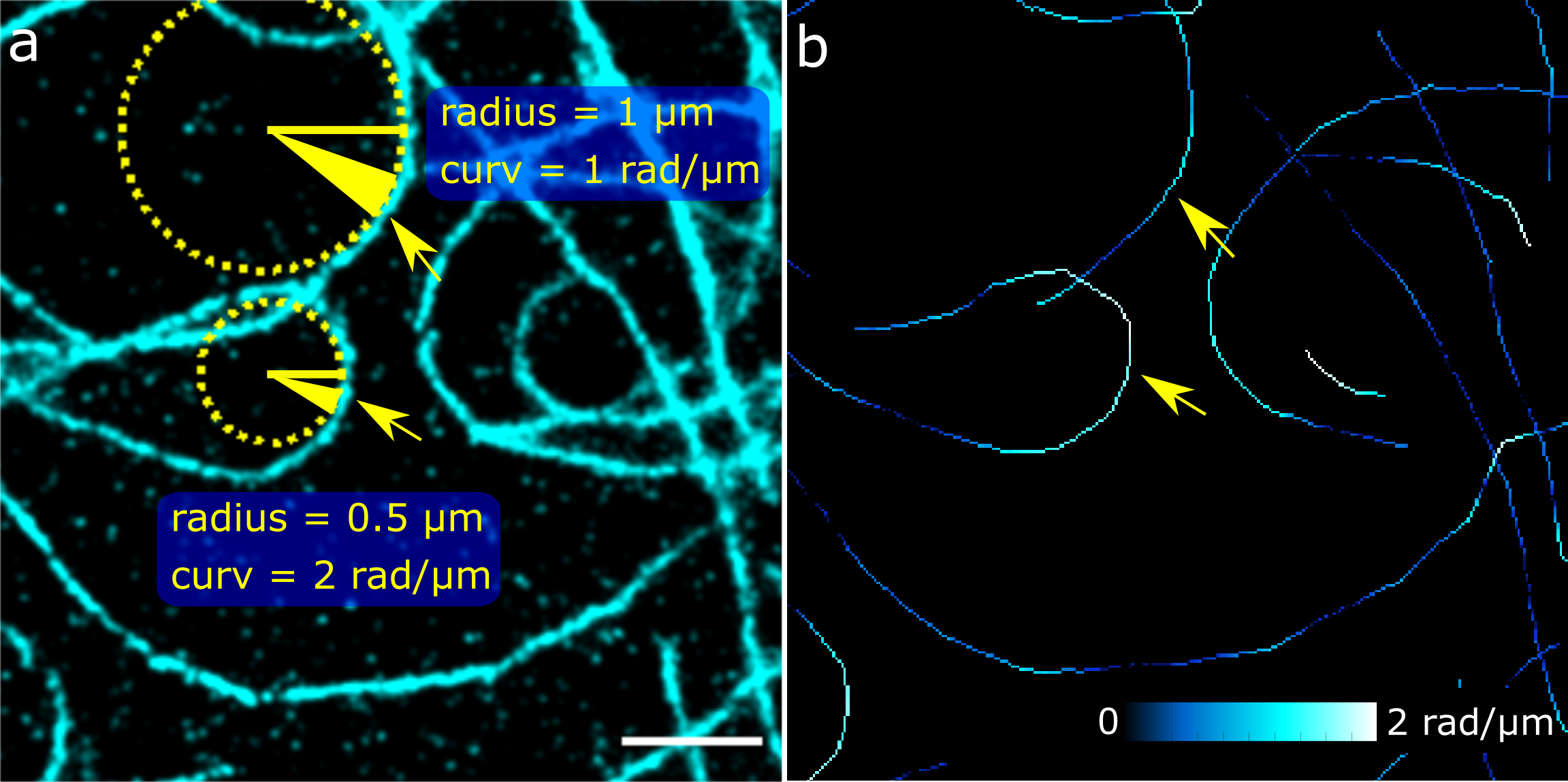


**Fig S3.** Filament curvature analysis from *d*STORM images using SIFNE. **(a)** *d*STORM image of filament curvature from 80 nM colcemid treated cell with annotated circles of 0.5 µm and 1 µm radius to visualize curvatures of 2 rad/µm and 1 rad/µm respectively. **(b)** Output from SIFNE of traced filaments colour coded for curvature. Curvatures indicated by yellow arrows match the curvature measurements shown in a.


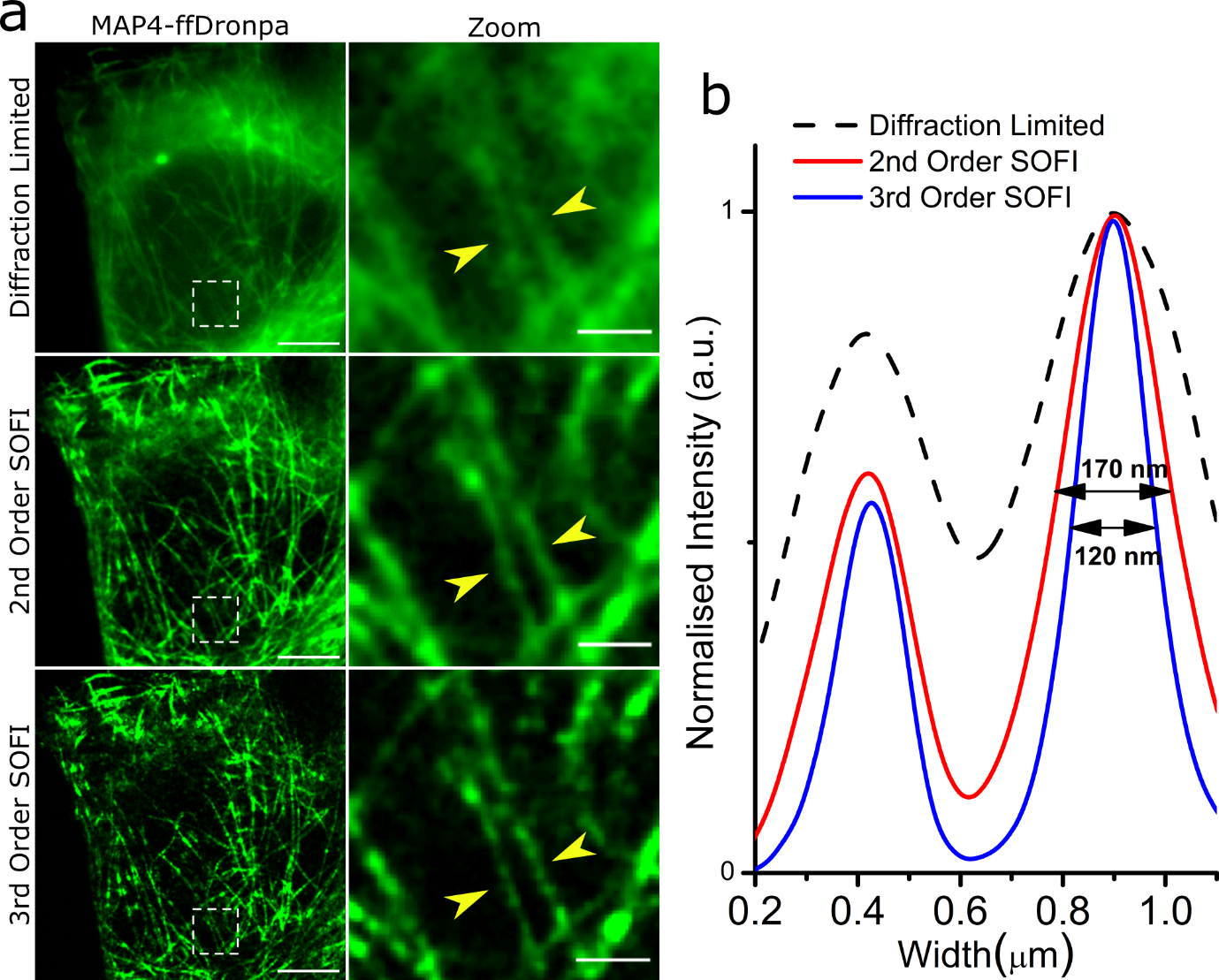


**Fig S4.** 2^nd^ and 3^rd^ order SOFI for subdiffraction resolution of live-cell HeLa MT filaments. **(a)** Imaging of representative live HeLa cell transfected with MAP4-ffDronpa, diffraction limited (top) and rendered with 2^nd^ order SOFI (middle) or 3^rd^ order SOFI (bottom). Zoomed regions (white boxes) show the same adjacent MT filaments from each image. Scale bars = 5 µm (left-hand images) and 1 µm (right-hand images). **(b)** Intensity cross-sections across MT filaments within yellow arrows for each zoomed region. Width values shown for filament visualised in 2^nd^ and 3^rd^ order SOFI are derived after fitting the intensity peak with a Gaussian.


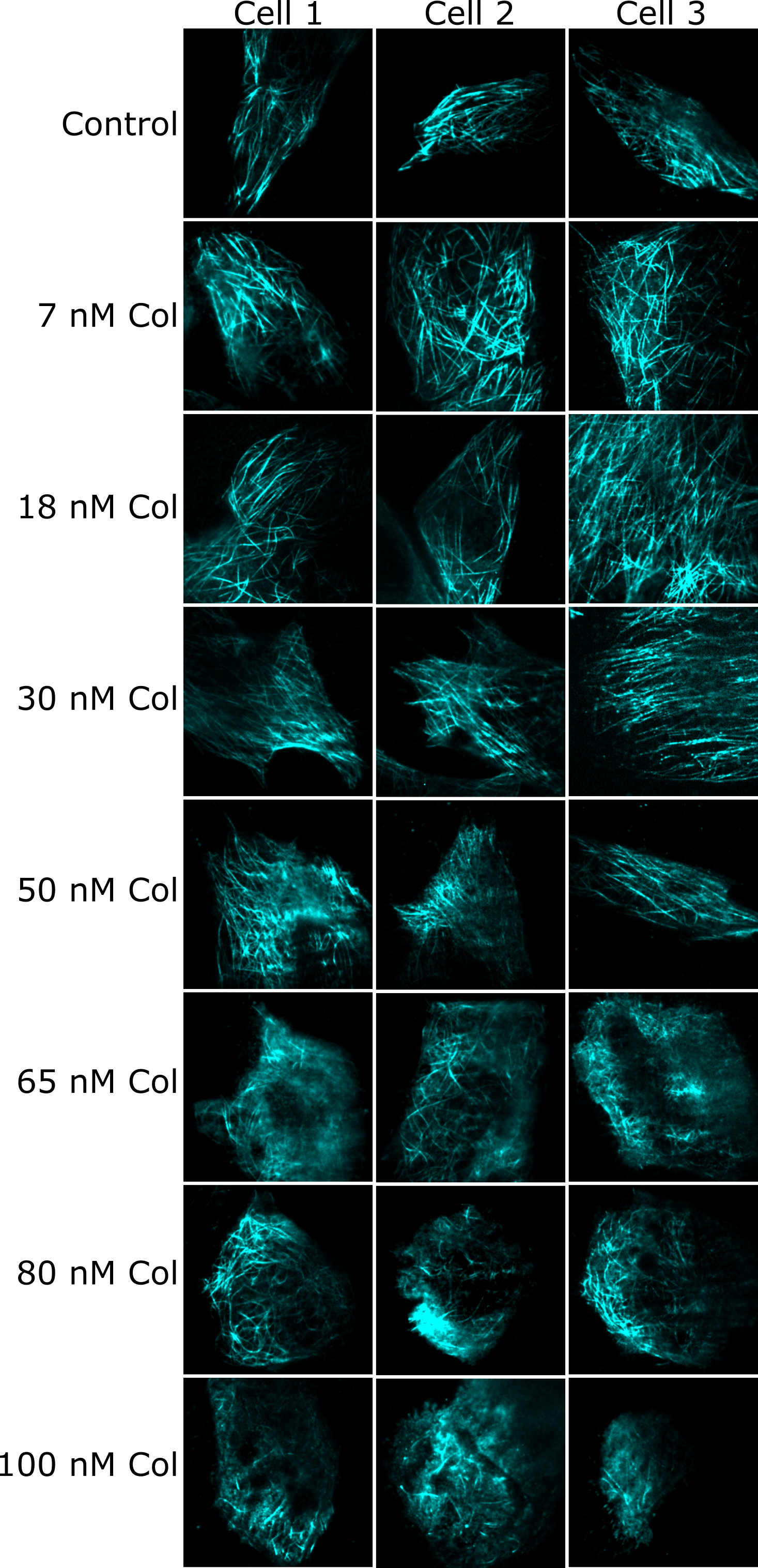


**Fig S5.** HeLa cells treated with colcemid and imaged with SOFI. Each frame is 15 µm X 15 µm.


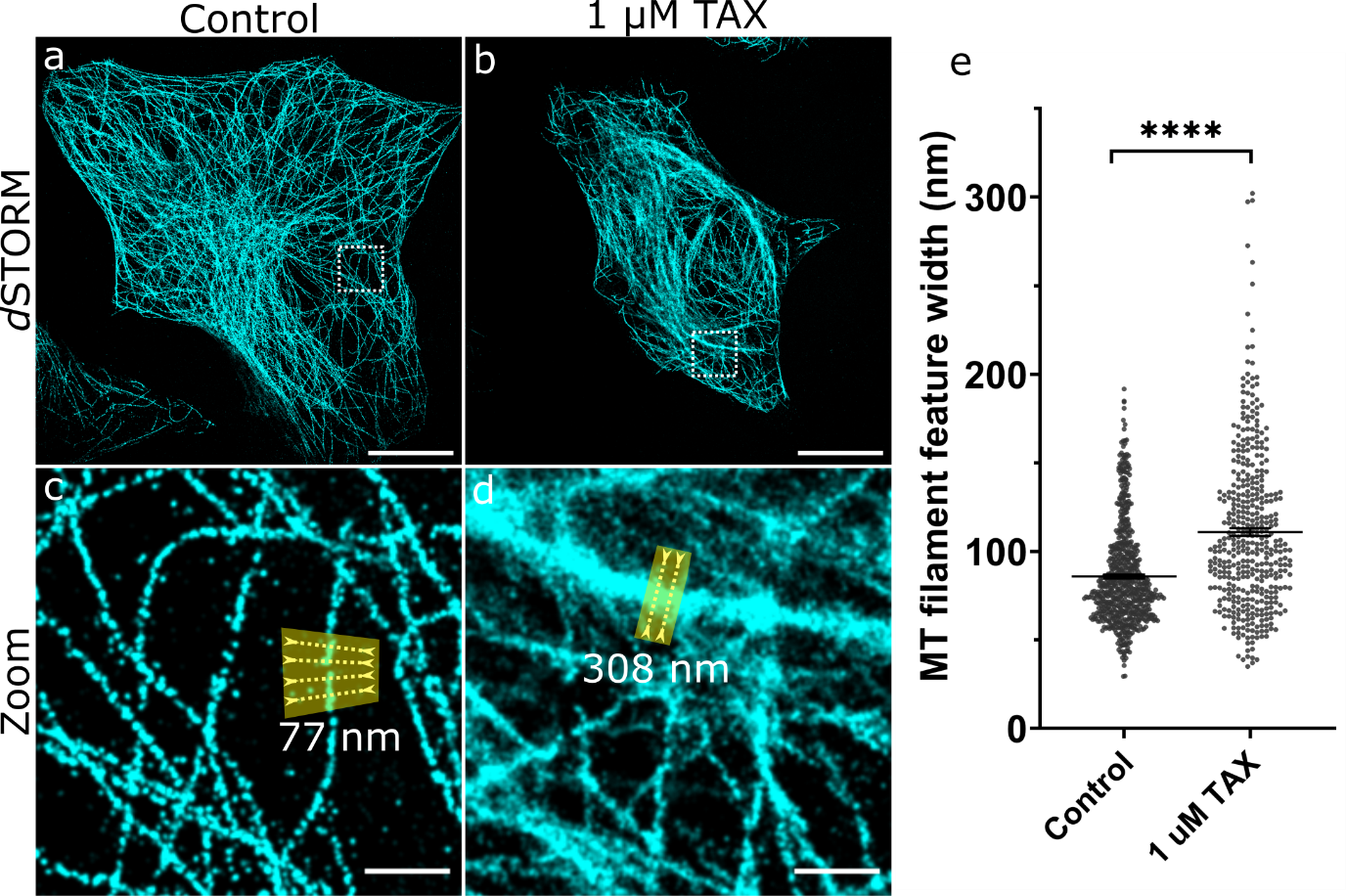
**Fig S6.** Application of *d*STORM to quantify MT bundling induced by paclitaxel (TAX). Representative images of HeLa cells untreated **(a)** or treated with 1 µM TAX for 5 hours **(b)** that were fixed (as previously described) and imaged using *d*STORM. **(c,d)** Zoomed regions from dotted white boxes in (a) and (b) respectively. Filaments were analysed using LineProfiler that traced filaments and measured multiple intensity cross-sections (dotted yellow lines) along a section of filament length (yellow box) ranging a few µm (~1 – 3 µm) to derive an average width based on a Gaussian fit of the combined cross-sections. **(e)** A combined plot of all the average widths measured from filament sections within a 400 µm^2^ area/cell (n = 5 cells, >400 average widths plotted) for each control and TAX treated cells. Plots show mean ± standard error of mean for control (86 ± 1 nm) and TAX treated (111 ± 2 nm). Parametric unpaired t-test revealed a significant increase (****, p < 0.0001) in the average MT filament feature width with 1 µM of TAX, indicative of TAX-induced MT bundling. Scale bars = 10 µm (a,b) and 1 µm (c,d). Data for e provided in Additional file 3: Sheet 4.
